# Plasma neurofilament light chain is associated with cognitive decline in non-dementia older adults

**DOI:** 10.1038/s41598-021-91038-0

**Published:** 2021-06-28

**Authors:** Lingxiao He, John E. Morley, Geetika Aggarwal, Andrew D. Nguyen, Bruno Vellas, Philipe de Souto Barreto, the MAPT/DSA Group, Sandrine Andrieu, Christelle Cantet, Nicola Coley

**Affiliations:** 1 Gérontopôle de Toulouse, Institut du Vieillissement, Centre Hospitalo-Universitaire de Toulouse, 37 Allées Jules Guesdes, 31000 Toulouse, France; 2 Division of Geriatric Medicine, Saint Louis University School of Medicine, St. Louis, MO USA; 3 Henry and Amelia Nasrallah Center for Neuroscience, Saint Louis University, St. Louis, MO USA; 4 CERPOP, Inserm 1295, Université de Toulouse, UPS, 31000 Toulouse, France; 5 CHU Toulouse, Toulouse, France; 6 University of Seattle, Seattle, USA; 7 University of Montreal, Montreal, Canada; 8 CHU de Bordeaux, Bordeaux, France; 9 Hospital of Castres, Castres, France; 10 CHU de Dijon, Dijon, France; 11 Hospital of Foix, Foix, France; 12 Hospital of Lavaur, Lavaur, France; 13 University Hospital of Limoges, Limoges, France; 14 Centre Hospitalier Lyon-Sud, Lyon, France; 15 Hospital of Princess Grace, La Colle, Monaco; 16 Hospital of Montauban, Montauban, France; 17 University Hospital of Montpellier, Montpellier, France; 18 University Côte d’Azur, Nice, France; 19 Hospital of Tarbes, Tarbes, France; 20 CATI Multicenter Neuroimaging Platform, Gif-sur-Yvette, France

**Keywords:** Predictive markers, Neurological disorders

## Abstract

Neurofilament light chain (NfL) has been associated with cognitive status in multiple neurodegenerative conditions. Studies about plasma NfL and cognitive decline in older adults are still limited. 504 older adults (median age 75 years) who expressed memory complaints were selected from the Multidomain Alzheimer’s Preventive Trial (MAPT) and were classified as normal cognition (NC) or mild cognitive impairment (MCI). Cognitive functions were measured as mini mental state examination (MMSE) and composite cognitive score (CCS) over a 4-year period. Plasma NfL was measured at the first or the second year of the MAPT. Mixed-effects linear models were performed to evaluate cross-sectional and longitudinal associations. In the whole population, higher plasma NfL was cross-sectionally associated with lower cognitive functions (MMSE: β =  − 0.007, 95% CI [− 0.013, − 0.001]; CCS: β =  − 0.003, 95% CI [− 0.006, − 0.001]). In adults with MCI, but not NC, higher plasma NfL was associated with lower CCS at the cross-sectional level (β =  − 0.003, 95% CI [− 0.005, − 0.0002]). The upper quartile NfL group further demonstrated more over time decline in CCS (β =  − 0.07, 95% CI [− 0.12, − 0.01]) under the MCI status. Plasma NfL can be a promising biomarker of progressive cognition decline in older adults with MCI.

## Introduction

Neurofilament is a structural protein that determines axonal caliber and conduction velocity in neurons^1^. As one of the three neurofilament components, neurofilament light chain (NfL) has been suggested as a biomarker of axonal damage^2^. High cerebrospinal fluid (CSF) NfL levels have been found in patients with multiple neurodegenerative conditions such as Parkinson’s disease^3^, Alzheimer’s disease (AD)^4^ and frontotemporal dementia^5^. As an inexpensive and less invasive parameter, plasma NfL is supposed to be a substitute of CSF NfL in the evaluation of neural degeneration due to their close correlations^6^. Elevated plasma and CSF NfL levels are found in multiple neural degenerative disorders such as mild cognitive impairment (MCI)^7^, AD^8,9^ and amyotrophic lateral sclerosis^10^. Plasma NfL also demonstrated similar effect sizes as CSF NfL in the association with over time cognitive declines among older adults (with a median age of 76)^7^. Cross-sectional studies have shown that older adults with MCI had higher plasma NfL than those with normal cognition (NC), and higher NfL was associated with lower cognitive functions^8,11^. At a longitudinal level, greater baseline plasma NfL was associated with poorer cognitive functions over time^7,8^.

Despite these findings, cross-sectional and longitudinal studies of plasma NfL and cognitive functions in non-dementia older adults are still limited, and with relatively small sample sizes. Contradictory results about the significance of plasma NfL difference between the NC and MCI groups were also reported in various studies^7,8,11,12^. Moreover, although plasma NfL has been associated with both neuroimaging measures (e.g., hippocampal volume and cortical thickness) and cognitive functions^7^, it is not known if such NfL-cognition association is mediated by the brain structures.

Therefore, the aim of this study is to explore both cross-sectional and longitudinal associations between plasma NfL levels and cognition in non-dementia community-dwelling older adults with a large sample size. Further exploratory analysis was performed to explore whether the associations between plasma NfL and cognitive functions were mediated by brain imaging neurodegeneration markers (e.g., white matter hyperintensities, hippocampal volume).

## Results

Descriptive data of the participants are presented in Table 1. Among the 504 participants (median age 75 years) included in this study, 60% were female and the median plasma NfL level was 72.9 pg/ml (with an interquartile range [IQR] of 56.9 to 91.8 pg/ml). The MCI group included 281 participants and the NC group included 223 participants. The MCI group showed slightly higher plasma NfL levels than the NC group, but the difference was not significant. The MCI group were older than the NC group and demonstrated lower cognitive scores (Table 1). In the MCI group, the median value of initial mini mental state examination (MMSE) was 28 (IQR: [26, 29]) and the median value of initial composite cognitive score (CCS) was -0.04 (IQR: [− 0.61, 0.48]). In the NC group, the median value of initial MMSE was 29 (IQR: [28, 30]) and the median value of initial CCS was 0.37 (IQR: [0.01, 0.70]).

**Table 1:** Descriptive data.

	Whole population		NC group		MCI group	
	Sample size	N (%) or Mean (SD) or Median [P25, P75]	Sample size	N (%) or Mean (SD) or Median [P25, P75]	Sample size	N (%) or Mean (SD) or Median [P25, P75]
Female	504	303 (60%)	223	145 (65%)	281	158 (56%)*
**MAPT groups**						
Multidomain training + omega-3 supplementation	504	129 (26%)	223	49 (22%)	281	80 (28%)
Omega-3 supplementation	504	118 (23%)	223	53 (24%)	281	65 (23%)
Multidomain training	504	124 (25%)	223	55 (25%)	281	69 (25%)
Placebo	504	133 (26%)	223	66 (30%)	281	67 (24%)
Participants had NfL tested from first year blood samples	504	465 (92%)	223	213 (96%)	281	252 (90%)
Age	504	75.0 [72.0, 79.0]	223	74.0 [71.0, 78.0]	281	76.0 [72.0, 79.0]*
Initial BMI	501	26.0 [23.7, 28.7]	220	26.1 [24.0, 29.2]	281	25.9 [23.4, 28.1]
Initial plasma NfL (pg/ml)	504	72.9 [56.9, 91.8]	223	71.8 [56.7, 89.7]	281	72.9 [56.9, 93.9]
Initial MMSE	502	28.0 [27.0, 79.0]	223	29.0 [28.0, 30.0]	279	28.0 [26.0, 29.0]**
Initial MMSE orientation	502	10.0 [10.0, 10.0]	223	10.0 [10.0, 10.0]	279	10.0 [9.0, 10.0]**
Initial FCSRT	501	76.0 [70.0, 81.0]	223	79.0 [74.0, 83.0]	278	73.0 [66.0, 79.0]**
Initial DSST-WAISR	499	37.5 (10.1)	223	39.6 (10.2)	276	36.0 (13.5)**
Initial category naming	501	25.5 (7.7)	223	27.2 (7.1)	278	24.0 (10.0)**
Initial CCS	499	0.17 [-0.27, 0.55]	223	0.37 [0.01, 0.70]	276	− 0.04 [− 0.61, 0.48]**

*BMI* body mass index, *CCS* composite cognitive score, *FCSRT* free and cued selective reminding test, *NC* normal cognition (CDR = 0), *MCI* mild cognitive impairment (CDR = 0.5), *MMSE* mini–mental state examination, *NfL* neurofilament light chain, *DSST-WAISR* Digit Symbol Substitution Test score from the Wechsler Adult Intelligence Scale—Revised.

*p < 0.05, **p < 0.01 compared with the NC group.

**Figure 1:**
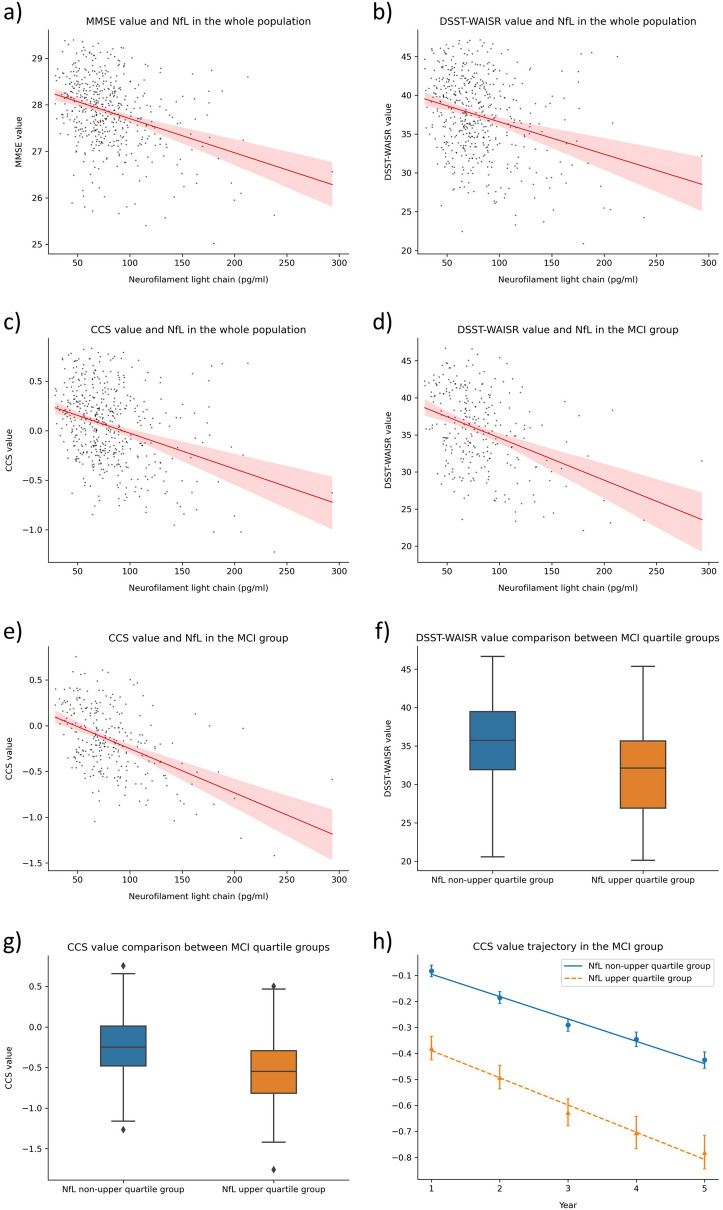
Significant findings in mixed-effects models. (**a**) Correlation between MMSE and NfL in the whole population at the initial level. (**b**) Correlation between DSST-WAISR and NfL in the whole population at the initial level. (**c**) Correlation between CCS and NfL in the whole population at the initial level. (**d**) Correlation between DSST-WAISR and NfL in the MCI group at the initial level. (**e**) Correlation between CCS and NfL in the MCI group at the initial level. (**f**) DSST-WAISR comparison between quartile groups in the MCI group at the initial level. (**g**) CCS comparison between quartile groups in the MCI group at the initial level. (**h**) CCS trajectory in the MCI group over a 4-year period (standard errors are used as error bars).

At the cross-sectional level, plasma NfL demonstrated negative association with MMSE (β =  − 0.007, 95% CI [− 0.013, − 0.001], Fig. 1a), digit symbol substitution test of the Wechsler adult intelligence scale-revised (DSST-WAISR) (β =  − 0.04, 95% CI [− 0.07, − 0.01], Fig. 1b) and CCS (β =  − 0.003, 95% CI [− 0.006, − 0.001], Fig. 1c) in the whole population (Table 2), but no association was found in the NC group (Table 2). In the MCI group, higher plasma NfL levels were associated with lower DSST-WAISR (β =  − 0.04, 95% CI [− 0.07, − 0.01], Fig. 1d) and CCS scores (β =  − 0.003, 95% CI [− 0.005, − 0.0002], Fig. 1e) (Table 2). No longitudinal associations were found between plasma NfL and cognitive functions in either cognitive groups.

**Table 2:** Mixed-effects linear analysis of plasma NfL with cognitive functions.

	Sample size	Initial NfL			Time			Initial NfL × time		
		Coefficient	*p*	95% CI	Coefficient	*p*	95% CI	Coefficient	*p*	95% CI
**Whole population**										
MMSE	496	− 0.007	0.02	(− 0.013, − 0.001)	− 0.129	0.06	(− 0.260, 0.003)	0.0005	0.54	(− 0.0011, 0.0021)
MMSE orientation	496	− 0.002	0.06	(− 0.004, 0.0001)	− 0.019	0.42	(− 0.067, 0.028)	− 0.0001	0.75	(− 0.0007, 0.0005)
FCSRT	496	− 0.03	0.11	(− 0.07, 0.01)	− 0.12	< 0.001	(− 1.84, − 0.56)	− 0.0001	0.98	(− 0.008, 0.008)
DSST-WAISR	496	− 0.04	0.01	(− 0.07, − 0.01)	− 0.67	0.01	(− 1.14, − 0.19)	− 0.001	0.78	(− 0.006, 0.005)
Category naming	496	− 0.02	0.20	(− 0.04, 0.01)	− 0.48	0.04	(− 0.93, − 0.02)	− 0.002	0.46	(− 0.007, 0.003)
CCS	496	− 0.003	0.006	(− 0.006, − 0.001)	− 0.07	0.001	(− 0.11, − 0.03)	− 0.0003	0.33	(− 0.0008, 0.0003)
**NC group**										
MMSE	219	− 0.006	0.08	(− 0.013, 0.001)	− 0.215	0.02	(− 0.389, − 0.042)	0.0015	0.13	(− 0.0005, 0.0036)
MMSE orientation	219	− 0.001	0.29	(− 0.003, 0.001)	− 0.04	0.13	(− 0.09, 0.01)	0.0002	0.43	(− 0.0004, 0.0008)
FCSRT	219	− 0.01	0.58	(− 0.04, 0.02)	− 0.80	0.10	(− 1.75, 0.14)	− 0.002	0.78	(− 0.013, 0.009)
DSST-WAISR	219	− 0.002	0.94	(− 0.045, 0.042)	− 0.76	0.04	(− 1.49, − 0.03)	0.001	0.86	(− 0.008, 0.009)
Category naming	219	0.01	0.53	(− 0.02, 0.04)	− 0.55	0.12	(− 1.24, 0.14)	0.0002	0.96	(− 0.008, 0.008)
CCS	219	− 0.0003	0.80	(− 0.003, 0.002)	− 0.067	0.03	(− 0.126, − 0.007)	− 0.0001	0.73	(− 0.0008, 0.0006)
**MCI group**										
MMSE	277	− 0.005	0.16	(− 0.012, 0.002)	− 0.04	0.69	(− 0.24, 0.16)	− 0.001	0.55	(− 0.003, 0.002)
MMSE orientation	277	− 0.001	0.43	(− 0.004, 0.002)	− 0.003	0.93	(− 0.082, 0.075)	− 0.0004	0.43	(− 0.001, 0.001)
FCSRT	277	− 0.02	0.29	(− 0.07, 0.02)	− 1.51	0.001	(− 2.42, − 0.60)	0.0004	0.94	(− 0.011, 0.011)
DSST-WAISR	277	− 0.04	0.007	(− 0.07, − 0.01)	− 0.63	0.048	(− 1.25, − 0.01)	− 0.002	0.60	(− 0.010, 0.006)
Category naming	277	− 0.01	0.44	(− 0.04, 0.02)	− 0.32	0.29	(− 0.92, 0.28)	− 0.005	0.18	(− 0.012, 0.002)
CCS	277	− 0.003	0.04	(− 0.005, − 0.0002)	− 0.07	0.02	(− 0.14, − 0.01)	− 0.0004	0.25	(− 0.001, 0.0003)

*FCSRT* Free and cued selective reminding test, *DSST-WAISR* Digit Symbol Substitution Test score from the Wechsler Adult Intelligence Scale—Revised.

Analysis using stratified NfL failed to find any cross-sectional or longitudinal association between NfL and cognitive functions in the NC group (Table 3). In MCI, the NfL+ subgroup showed lower DSST-WAISR (β =  − 3.62, 95% CI [− 6.1, − 1.14], Fig. 1f) and CCS scores (β =  − 0.21, 95% CI [− 0.42, − 0.01], Fig. 1g) at the cross-sectional level, and greater over time decline in the CCS score than the NfL− subgroup (Table 3, Fig. 1h).

**Table 3:** Mixed-effects linear analysis of plasma NfL with cognitive functions in stratified NfL quartile subgroups.

	Sample size	NfL+ group^#^			Time			NfL+ group^#^ × time		
		Coefficient	*p*	95% CI	Coefficient	*p*	95% CI	Coefficient	*p*	95% CI
**NC group**										
MMSE	219	− 0.35	0.21	(− 0.90, 0.19)	− 0.04	0.60	(− 0.19, 0.11)	0.07	0.41	(− 0.10, 0.24)
MMSE orientation	219	− 0.01	0.86	(− 0.16, 0.13)	− 0.03	0.16	(− 0.07, 0.01)	− 0.01	0.61	(− 0.06, 0.04)
FCSRT	219	− 0.91	0.48	(− 3.43, 1.61)	− 0.92	0.02	(− 1.72, − 0.13)	0.005	0.99	(− 0.89, 0.90)
DSST-WAISR	219	− 1.18	0.49	(− 4.56, 2.20)	− 0.79	0.01	(− 1.40, − 0.17)	− 0.12	0.74	(− 0.81, 0.58)
Category naming	219	− 0.07	0.96	(− 2.37, 2.24)	− 0.52	0.08	(− 1.11, 0.06)	0.01	0.97	(− 0.65, 0.67)
CCS	219	− 0.05	0.63	(− 0.23, 0.14)	− 0.10	< 0.001	(− 0.15, − 0.05)	− 0.03	0.33	(− 0.08, 0.03)
**MCI group**										
MMSE	277	− 0.42	0.15	(− 0.99, 0.15)	− 0.14	0.10	(− 0.31, 0.03)	− 0.05	0.57	(− 0.24, 0.13)
MMSE orientation	277	− 0.01	0.94	(− 0.20, 0.19)	− 0.09	0.01	(− 0.15, − 0.02)	− 0.07	0.07	(− 0.14, 0.01)
FCSRT	277	− 2.23	0.22	(− 5.81, 1.34)	− 1.76	< 0.001	(− 2.51, − 1.02)	− 0.37	0.38	(− 1.20, 0.46)
DSST-WAISR	277	− 3.62	0.004	(− 6.10, − 1.14)	− 0.94	< 0.001	(− 1.44, − 0.43)	− 0.20	0.49	(− 0.77, 0.37)
Category naming	277	− 1.70	0.13	(− 3.88, 0.49)	− 0.84	< 0.001	(− 1.32, − 0.35)	− 0.17	0.54	(− 0.72, 0.37)
CCS	277	− 0.21	0.04	(− 0.42, − 0.01)	− 0.16	< 0.001	(− 0.21, − 0.11)	− 0.07	0.02	(− 0.12, − 0.01)

*FCSRT* free and cued selective reminding test, *DSST-WAISR* Digit Symbol Substitution Test score from the Wechsler Adult Intelligence Scale—Revised.

^#^Participants in the non-upper quartile (NfL−) group as the reference.

Additionally, after adjusting for the APOE genotype, only marginal significance was found at the cross-sectional level between NfL and CCS (in the whole population and the NC group) while in the MCI group, significant cross-sectional association was found between NfL and DSST-WAISR (β =  − 0.04, 95% CI [− 0.07, − 0.01]). No significant longitudinal NfL association with cognitive functions were found in models with the APOE genotype (Supplementary File Table 3 and 4). We further replicated the analyses only on the control group and similar results were found in the whole control group (β =  − 0.07, 95% CI [− 0.13, − 0.01]) and the MCI control group (β =  − 0.08, 95% CI [− 0.14, − 0.02]) that higher plasma NfL levels were cross-sectionally associated with lower DSST-WAISR scores (Supplementary File Table 5 and 6).

Exploratory mediation analysis (n = 176) was further performed using the structural equation modelling (SEM) method (Supplementary File Table 7 and 8) and demonstrated significant direct effects of plasma NfL on DSST-WAISR and CCS in the whole population (n = 176), and on DSST-WAISR in the MCI population (n = 85), while no indirect effects (mediated by white matter condition and cognition-related brain structures) were found.

## Discussion

The present study examined cross-sectional and longitudinal associations between plasma NfL and cognitive functions in non-dementia community-dwelling older adults (aged 70 years or above). Among adults without cognitive impairment (i.e., the NC group), plasma NfL was not associated with cognitive functions. In the MCI population, higher plasma NfL was associated with lower global cognitive scores (i.e., CCS) and executive function (i.e., DSST-WAISR) at a cross-sectional level. Moreover, MCI adults with upper quartile NfL levels demonstrated greater over time declines in the CCS score. Exploratory analysis using SEM failed to find any mediation effect of brain structures. Together, our results support the idea of using plasma NfL as a marker of predicting cognitive decline in MCI individuals^13^.

Based on the significant associations found in the main analysis, we further performed an exploratory mediation analysis using the SEM method which failed to detect any mediation effect of neuroimaging measures in the association between plasma NfL and cognitive function. Since a previous study by Mielke et al.^7^ also failed to find cross-sectional association between baseline plasma NfL with cortical thickness and hippocampal volume, the cross-sectional association between plasma NfL and cognitive functions might not be mediated by brain structures. A possible explanation might be that our participants were still in an early phase of neurodegeneration when increased plasma NfL could be found while no obvious brain structural changes could be detected.

We found that plasma NfL was significantly associated with cognitive functions only in the MCI group. Such result is in line with the findings of Mattsson et al.^8^, who studied plasma NfL and cognition in the NC, MCI and AD populations and reported that plasma NfL was related to longitudinal changes of MMSE only in the MCI adults. A possible explanation for the specific association found only in the MCI group might be that there are larger variances in plasma NfL and cognitive functions in the MCI population than the NC one^8,11,12^, making the plasma NfL association with cognitive functions much easier to be detected.

The large sample size and the longitudinal cognitive data over a 4-year period are the strengths of our study. Notably, the differences of cognitive functions between the NC and the MCI group were bigger in subjects of the Multidomain Alzheimer’s Preventive Trial (MAPT) cohort compared to the current study (i.e., cognitive functions of the NC group in the current study were lower than the larger MAPT cohort [Supplementary File Table 1], the CCS in the MCI group was higher in the current study than that in the MAPT [Supplementary File Table 2]). Additionally, we attempted to combine brain imaging data to explore the mediation effect of brain structures on the NfL and cognition relationship. However, this study is limited by the lack of longitudinal plasma NfL data, impeding us from examining a time-matched association between the plasma NfL and cognitive functions. Moreover, since the plasma NfL was tested during the MAPT intervention, we do not know if the interventions have caused any adaptive changes in plasma NfL. Although we have adjusted the MAPT groups in our analysis, further cohort studies with baseline NfL data are needed to generalize our findings to a wider population. Moreover, besides NfL, other cognition-related blood biomarkers, such as brain-derived neurotrophic factor, 3-hydroxykynurenine, lipid levels and total-tau^14,15^, might also be tested as potential candidates in similar analysis to distinguish cognitive status between the NC and the MCI groups. Despite that we found associations between NfL and global cognitive functions in the MCI group, investigations using a more comprehensive assessment of cognitive functions^7,11,16^ is needed to confirm our findings.

To conclude, the current study analyzed the association between plasma NfL and cognitive function in non-dementia older adults over an up to 4-year period. We found that the plasma NfL was not associated with cognitive functions in the NC adults while higher plasma NfL levels were associated with lower CCS and more CCS decline in the MCI adults. Our results suggested the possibility of using plasma NfL as a marker of predicting cognitive decline in MCI individuals. Future studies on the mechanism between plasma NfL and cognitive functions are still needed.

## Methods

### Study population

Participants in this study came from the randomized controlled trial MAPT (ClinicalTrials.gov [NCT00672685]), which examined 1679 dementia-free older adults (aged ≥ 70 years). These adults were recruited with any of the following criteria: (1) expressing spontaneous memory complaint, (2) having limitation in at least one instrumental activity of daily, (3) demonstrating slow gait speed (i.e., lower than 0.8 m/s). Participants were excluded if any of the following criteria was met: (1) MMSE score ≤ 24, (2) diagnosed dementia, (3) having difficulties in basic activities of daily living, (4) taking polyunsaturated fatty acid supplementation. The MAPT tested multidomain interventions (physical activity, nutritional counselling and cognitive training) and omega-3 supplementation, combined or alone, against placebo among older adults and examined changes in cognitive functions over a 3-year period^17^. The participants were further observationally followed for two additional years, without receiving any intervention. The MAPT was approved by the ethics committee in Toulouse (CPP SOOM II). Written consent forms were obtained from all participants. All research was performed in accordance with relevant guidelines/regulations.

In the current study, participants with extreme plasma NfL levels (n = 5, over four standard deviations [SDs] above the mean value) or with no Clinical Dementia Rating (CDR, n = 3) score were excluded from an initial group (n = 512) in the MAPT that had received plasma NfL tests. Among the 504 participants included in the current study, those with a CDR score of 0.5 were defined as having MCI while others with NC had a CDR score of 0.

### Measurement of plasma NfL

Blood samples were stored in EDTA coated tubes. Plasma neurofilament light chain levels were determined by an electrochemiluminescence-based assay using the R-PLEX human neurofilament L antibody set (F217X-3) with MSD Gold 96-well Small Spot SA SECTOR plates (L45A-1). Samples were diluted twofold in Diluent 12 (R50JA-3) and assayed in duplicate, and read with a Meso Scale Discovery instrument. The mean intra-assay coefficient of variation was 7.8%, and the inter-assay coefficient of variation between plates was 15.4%. Over 90% of the participants (n = 465) had plasma NfL tested from blood samples taken 1 year after the enrolment in the study. For the rest of the participants, blood samples from the second year were used. Blood sampling was performed on the same day of cognitive tests.

### Measurement of neuroimaging variables

The magnetic resonance imaging (MRI) scan was performed within 12 months after MAPT enrollment^17^. The 3D T1-weighted sequence, derived by the SPM5 toolbox (fil.ion.ucl.ac.uk/spm), was used to measure MRI images. In this study, white matter volume (cm^3^), white matter hyperintensities, hippocampal volume (cm^3^) and amygdala volume (cm^3^) were used for exploratory analysis.

### Outcome measures—cognitive functions

Participants completed a comprehensive assessment of four domains: memory (free and total recall of the Free and Cued Selective Reminding Test [FCSRT]), language (the Category Naming Test), executive function (the DSST-WAISR) and orientation (ten MMSE orientation items)^18^.

Global cognitive functions were evaluated as MMSE (ranging from 0 to 30, higher is better) and CCS (mean z-scores of the four domains). In the calculation of CCS, the z-score of each domain was calculated using the initial mean and SD values of corresponding test. Original values of each cognitive test and global cognitive functions evaluated at the time-point and after blood tests were included for further analysis.

### Covariates

Data of age, sex, body mass index (BMI), education level and MAPT group (i.e., multidomain training + omega-3 supplementation group, multidomain training group, omega-3 supplementation group and control group) were collected and were controlled in the analyses of this study.

### Statistics

Descriptive data are presented as mean ± SD or median [interquartile range] or frequency (percentage), and comparisons between the NC and the MCI groups were performed using Student’s t-test, Wilcoxon rank-sum test or chi-squared test as appropriate. Mixed-effects linear models were performed to analyze the cross-sectional and longitudinal associations between plasma NfL and cognitive functions in the whole population and by each cognitive group (i.e., NC and MCI groups). Random effect of participants and random slope of time were assumed.

Within each cognitive group, we further stratified participants into an NfL upper quartile (NfL+) group and a non-upper quartile (NfL−) group with upper quartile NfL values as the cut-off values (89.7 pg/ml in the NC group and 93.86 pg/ml in the MCI group). Similar mixed-effects linear models were performed with stratified NfL groups as an independent variable. Analyses were performed using SAS 9.4 with a two-sided significance level of 0.05. Result visualization was performed by Python 3.7^19^. Due to a large amount of missing values (i.e., 10% of the present sample), APOE4 was not included in the main model. Sensitive analyses were performed with the APOE genotype as an extra covariate.

Exploratory mediation analysis was performed based on 176 participants (85 MCI participants) with available data using SEM to explore whether the cross-sectional associations between plasma NfL (measured in the first year after the enrollment) and cognitive functions (measured in the first year) were mediated by white matter condition and cognition-related brain structures (measured at a baseline level). Baseline brain imaging neurodegeneration markers (i.e., white matter hyperintensities, white matter volume, hippocampus volume and amygdala volume) were used to create a latent variable of brain degeneration. The analysis was controlled for age, first year BMI and MAPT intervention groups, and was performed in R (version 4.0.3)^20^. Details of the analysis are included in the supplementary file.

## Supplementary information

Supplementary Informations.

## Data Availability

Data in this study are available upon request.
