# Supplementary material for: Plasma neurofilament light chain is associated with cognitive decline in non-dementia older adults
Source: Sci Rep. 2021 Jun 28;11:13394. doi: 10.1038/s41598-021-91038-0 (PMC8238930; doi:10.1038/s41598-021-91038-0)
Supplement: Supplementary file 1 — Supplementary Informations. [file 41598_2021_91038_MOESM1_ESM.docx]

**TITLE:**

Plasma neurofilament light chain is associated with cognitive decline in non-dementia older adults

Lingxiao He, PhD^1^, John E. Morley, MBBCh^2^, Geetika Aggarwal, PhD^2,3^, Andrew D. Nguyen, PhD^2,3^, Bruno Vellas, MD^1,4^, Philipe de Souto Barreto, PhD^1,4^, For the MAPT/DSA Group*

*^1^ Gérontopôle de Toulouse, Institut du Vieillissement, Centre Hospitalo-Universitaire de Toulouse, 37 allées Jules Guesdes, 31000 Toulouse, France;*

*^2^ Division of Geriatric Medicine, Saint Louis University School of Medicine, St. Louis, MO, USA;*

*^3^ Henry and Amelia Nasrallah Center for Neuroscience, Saint Louis University, St. Louis, MO, USA;*

*^4^ CERPOP, Inserm 1295, Université de Toulouse, UPS, 31000 Toulouse, France*

** Members are listed in the acknowledgements*

Table 1 Characteristics of the NC groups in the current study and the whole MAPT

|  | NC group in the current study | |  | NC group in the MAPT | |
| --- | --- | --- | --- | --- | --- |
|  | Sample size | N (%) or  Mean (SD) or  Median [P25, P75] |  | Sample size | N (%) or  Mean (SD) or  Median [P25, P75] |
| Female | 223 | 145 (65%) |  | 712 | 488 (69%) |
| MAPT groups |  |  |  |  |  |
| Multidomain training+omega-3 supplementation | 223 | 49 (22%) |  | 712 | 163 (23%) |
| Omega-3 supplementation | 223 | 53 (24%) |  | 712 | 180 (25%) |
| Multidomain training | 223 | 55 (25%) |  | 712 | 179 (25%) |
| Placebo | 223 | 66 (30%) |  | 712 | 190 (27%) |
| Age | 223 | 74.0 [71.0, 78.0] |  | 712 | 74.0 [71.0, 77.0] |
| Initial BMI | 220 | 26.1 [24.0, 29.2] |  | 706 | 25.9 [23.3, 28.7] |
| Initial MMSE | 223 | 29.0 [28.0, 30.0] |  | 712 | 29.0 [28.0, 30.0] |
| Initial MMSE orientation | 223 | 10.0 [10.0, 10.0] |  | 712 | 10.0 [10.0, 10.0] |
| Initial FCSRT | 223 | 79.0 [74.0, 83.0] |  | 712 | 81.0 [76.0, 85.0]** |
| Initial DSST-WAISR | 223 | 39.6 (10.2) |  | 712 | 42.3 (10.2)** |
| Initial Category naming | 223 | 27.2 (7.1) |  | 712 | 28.4 (7.5)* |
| Initial CCS | 223 | 0.37 [0.01, 0.70] |  | 712 | 0.26 [-0.06, 0.63] |

* p < 0.05, ** p < 0.01 compared with the NC group

BMI: Body mass index; CCS: Composite cognitive score; FCSRT: Free and cued selective reminding test; NC: Normal cognition (CDR = 0); MCI: Mild cognitive impairment (CDR = 0.5); MMSE: Mini–mental state examination; NfL: Neurofilament light chain; DSST-WAISR: Digit Symbol Substitution Test score from the Wechsler Adult Intelligence Scale—Revised

Table 2 Characteristics of the MCI groups in the current study and the whole MAPT

|  | MCI group in the current study | |  | MCI group in the MAPT | |
| --- | --- | --- | --- | --- | --- |
|  | Sample size | N (%) or  Mean (SD) or  Median [P25, P75] |  | Sample size | N (%) or  Mean (SD) or  Median [P25, P75] |
| Female | 281 | 158 (56%) |  | 682 | 402 (59%) |
| MAPT groups |  |  |  |  |  |
| Multidomain training+omega-3 supplementation | 281 | 80 (28%) |  | 682 | 185 (27%) |
| Omega-3 supplementation | 281 | 65 (23%) |  | 682 | 162 (24%) |
| Multidomain training | 281 | 69 (25%) |  | 682 | 173 (25%) |
| Placebo | 281 | 67 (24%) |  | 682 | 162 (24%) |
| Age | 281 | 76.0 [72.0, 79.0] |  | 682 | 75.0 [72.0, 79.0] |
| Initial BMI | 281 | 25.9 [23.4, 28.1] |  | 680 | 26.0 [23.5, 28.3] |
| Initial MMSE | 279 | 28.0 [26.0, 29.0] |  | 677 | 28.0 [26.0, 29.0] |
| Initial MMSE orientation | 279 | 10.0 [9.0, 10.0] |  | 677 | 10.0 [10.0, 10.0] |
| Initial FCSRT | 278 | 73.0 [66.0, 79.0] |  | 675 | 74.0 [67.0, 80.0] |
| Initial DSST-WAISR | 276 | 36.0 (13.5) |  | 673 | 36.9 (10.3) |
| Initial Category naming | 278 | 24.0 (10.0) |  | 675 | 24.6 (7.8) |
| Initial CCS | 276 | -0.04 [-0.61, 0.48] |  | 671 | -0.17 [-0.67, 0.29]** |

* p < 0.05, ** p < 0.01 compared with the NC group

BMI: Body mass index; CCS: Composite cognitive score; FCSRT: Free and cued selective reminding test; NC: Normal cognition (CDR = 0); MCI: Mild cognitive impairment (CDR = 0.5); MMSE: Mini–mental state examination; NfL: Neurofilament light chain; DSST-WAISR: Digit Symbol Substitution Test score from the Wechsler Adult Intelligence Scale—Revised

Table 3 Mixed-effects linear analysis of plasma NfL with cognitive functions (Adjusted for APOE)

|  | Sample size | Initial NfL | | |  | Time | | |  | Initial NfL × Time | | |
| --- | --- | --- | --- | --- | --- | --- | --- | --- | --- | --- | --- | --- |
|  |  | Coefficient | p | 95% CI |  | Coefficient | p | 95% CI |  | Coefficient | p | 95% CI |
| Whole population |  |  |  |  |  |  |  |  |  |  |  |  |
| MMSE | 453 | -0.004 | 0.25 | (-0.010, 0.003) |  | -0.171 | 0.01 | (-0.305, 0.037) |  | 0.001 | 0.22 | (-0.0006, 0.0026) |
| MMSE orientation | 453 | -0.001 | 0.32 | (-0.003, 0.001) |  | -0.031 | 0.21 | (-0.079, 0.018) |  | -0.0001 | 0.75 | (-0.0005, 0.0006) |
| FCSRT | 453 | -0.02 | 0.18 | (-0.06, 0.01) |  | -0.12 | <0.001 | (-1.90, -0.59) |  | 0.0002 | 0.96 | (-0.008, 0.008) |
| DSST-WAISR | 453 | -0.04 | 0.02 | (-0.07, -0.005) |  | -0.65 | 0.01 | (-1.14, -0.16) |  | -0.001 | 0.70 | (-0.007, 0.005) |
| Category naming | 453 | -0.01 | 0.39 | (-0.04, 0.01) |  | -0.46 | 0.06 | (-0.93, 0.01) |  | -0.002 | 0.40 | (-0.008, 0.003) |
| CCS | 453 | -0.002 | 0.05 | (-0.005, 0.00001) |  | -0.08 | 0.001 | (-0.12, -0.03) |  | -0.0002 | 0.43 | (-0.0008, 0.0003) |
|  |  |  |  |  |  |  |  |  |  |  |  |  |
| NC group |  |  |  |  |  |  |  |  |  |  |  |  |
| MMSE | 201 | -0.007 | 0.05 | (-0.014, -0.001) |  | -0.237 | 0.01 | (-0.423, -0.052) |  | 0.0016 | 0.15 | (-0.0006, 0.0037) |
| MMSE orientation | 201 | -0.001 | 0.29 | (-0.003, 0.001) |  | -0.04 | 0.14 | (-0.09, 0.01) |  | 0.0002 | 0.45 | (-0.0004, 0.0008) |
| FCSRT | 201 | -0.01 | 0.47 | (-0.04, 0.02) |  | -0.80 | 0.12 | (-1.80, 0.20) |  | -0.002 | 0.69 | (-0.014, 0.009) |
| DSST-WAISR | 201 | -0.001 | 0.97 | (-0.045, 0.044) |  | -0.72 | 0.06 | (-1.48, 0.04) |  | 0.0002 | 0.96 | (-0.009, 0.009) |
| Category naming | 201 | 0.01 | 0.70 | (-0.02, 0.04) |  | -0.59 | 0.12 | (-1.32, 0.14) |  | -0.0001 | 0.99 | (-0.009, 0.008) |
| CCS | 201 | -0.0004 | 0.72 | (-0.003, 0.002) |  | -0.067 | 0.04 | (-0.131, -0.001) |  | -0.0002 | 0.59 | (-0.001, 0.001) |
|  |  |  |  |  |  |  |  |  |  |  |  |  |
| MCI group |  |  |  |  |  |  |  |  |  |  |  |  |
| MMSE | 252 | -0.003 | 0.47 | (-0.010, 0.005) |  | -0.110 | 0.29 | (-0.314, 0.095) |  | 0.0003 | 0.82 | (-0.002, 0.003) |
| MMSE orientation | 252 | -0.001 | 0.71 | (-0.003, 0.002) |  | -0.027 | 0.51 | (-0.107, 0.054) |  | -0.0001 | 0.90 | (-0.001, 0.001) |
| FCSRT | 252 | -0.02 | 0.50 | (-0.06, 0.03) |  | -1.51 | 0.001 | (-2.43, -0.60) |  | 0.0007 | 0.90 | (-0.010, 0.012) |
| DSST-WAISR | 252 | -0.04 | 0.03 | (-0.07, -0.004) |  | -0.63 | 0.05 | (-1.28, 0.01) |  | -0.002 | 0.57 | (-0.010, 0.006) |
| Category naming | 252 | -0.005 | 0.75 | (-0.03, 0.02) |  | -0.28 | 0.38 | (-0.90, 0.34) |  | -0.005 | 0.17 | (-0.013, 0.002) |
| CCS | 252 | -0.002 | 0.14 | (-0.005, 0.001) |  | -0.08 | 0.01 | (-0.14, -0.02) |  | -0.0003 | 0.46 | (-0.001, 0.0005) |

FCSRT: Free and cued selective reminding test; DSST-WAISR: Digit Symbol Substitution Test score from the Wechsler Adult Intelligence Scale—Revised

Table 4 Mixed-effects linear analysis of plasma NfL with cognitive functions in stratified NfL quartile subgroups (Adjusted for APOE)

|  | Sample size | NfL+ group^#^ | | |  | Time | | |  | NfL+ group^#^ × Time | | |
| --- | --- | --- | --- | --- | --- | --- | --- | --- | --- | --- | --- | --- |
|  |  | Coefficient | p | 95% CI |  | Coefficient | p | 95% CI |  | Coefficient | p | 95% CI |
| NC group |  |  |  |  |  |  |  |  |  |  |  |  |
| MMSE | 201 | -0.39 | 0.16 | (-0.95, 0.16) |  | -0.05 | 0.50 | (-0.21, 0.10) |  | 0.07 | 0.41 | (-0.10, 0.25) |
| MMSE orientation | 201 | -0.01 | 0.94 | (-0.15, 0.14) |  | -0.03 | 0.15 | (-0.01, 0.08) |  | -0.02 | 0.55 | (-0.07, 0.03) |
| FCSRT | 201 | -0.75 | 0.57 | (-3.34, 1.84) |  | -1.05 | 0.01 | (-1.89, -0.21) |  | -0.08 | 0.86 | (-1.03, 0.87) |
| DSST-WAISR | 201 | -0.88 | 0.62 | (-4.36, 2.60) |  | -0.82 | 0.01 | (-1.46, -0.18) |  | -0.15 | 0.42 | (-0.88, 0.57) |
| Category naming | 201 | -0.05 | 0.97 | (-2.43, 2.33) |  | -0.60 | 0.06 | (-1.22, 0.02) |  | -0.01 | 0.99 | (-0.70, 0.69) |
| CCS | 201 | -0.03 | 0.79 | (-0.22, 0.16) |  | -0.11 | <0.001 | (-0.17, -0.06) |  | -0.04 | 0.26 | (-0.10, 0.03) |
|  |  |  |  |  |  |  |  |  |  |  |  |  |
| MCI group |  |  |  |  |  |  |  |  |  |  |  |  |
| MMSE | 252 | -0.42 | 0.17 | (-1.02, 0.18) |  | -0.04 | 0.68 | (-0.21, 0.13) |  | 0.07 | 0.49 | (-0.12, 0.26) |
| MMSE orientation | 252 | 0.02 | 0.86 | (-0.19, 0.22) |  | -0.06 | 0.08 | (-0.13, 0.01) |  | -0.03 | 0.37 | (-0.11, 0.04) |
| FCSRT | 252 | -1.40 | 0.45 | (-5.06, 2.27) |  | -1.61 | <0.001 | (-2.51, -1.02) |  | -0.20 | 0.65 | (-1.05, 0.65) |
| DSST-WAISR | 252 | -3.59 | 0.007 | (-6.20, -0.98) |  | -0.90 | 0.001 | (-1.43, -0.36) |  | -0.11 | 0.71 | (-0.71, 0.48) |
| Category naming | 252 | -1.38 | 0.23 | (-3.67, 0.90) |  | -0.84 | 0.002 | (-1.37, -0.33) |  | -0.21 | 0.49 | (-0.79, 0.38) |
| CCS | 252 | -0.18 | 0.10 | (-0.38, 0.03) |  | -0.14 | <0.001 | (-0.19, -0.09) |  | -0.04 | 0.14 | (-0.10, 0.01) |

^#^ Participants in the non-upper quartile (NfL-) group as the reference

FCSRT: Free and cued selective reminding test; DSST-WAISR: Digit Symbol Substitution Test score from the Wechsler Adult Intelligence Scale—Revised

Table 5 Mixed-effects linear analysis of plasma NfL with cognitive functions (analysis on MAPT control group only)

|  | Sample size | Initial NfL | | |  | Time | | |  | Initial NfL × Time | | |
| --- | --- | --- | --- | --- | --- | --- | --- | --- | --- | --- | --- | --- |
|  |  | Coefficient | p | 95% CI |  | Coefficient | p | 95% CI |  | Coefficient | p | 95% CI |
| Whole population |  |  |  |  |  |  |  |  |  |  |  |  |
| MMSE | 132 | -0.001 | 0.91 | (-0.011, 0.010) |  | -0.250 | 0.05 | (-0.497, -0.002) |  | 0.002 | 0.26 | (-0.001, 0.005) |
| MMSE orientation | 132 | -0.001 | 0.57 | (-0.006, 0.003) |  | -0.022 | 0.69 | (-0.128, 0.085) |  | -3.9E-6 | 0.99 | (-0.001, 0.001) |
| FCSRT | 132 | -0.038 | 0.32 | (-0.114, 0.038) |  | -0.193 | 0.004 | (-3.221, -0.641) |  | 0.01 | 0.19 | (-0.005, 0.026) |
| DSST-WAISR | 132 | -0.067 | 0.03 | (-0.128, -0.007) |  | -1.324 | 0.004 | (-2.222, -0.427) |  | 0.009 | 0.12 | (-0.002, 0.019) |
| Category naming | 132 | 0.01 | 0.67 | (-0.036, 0.056) |  | -0.079 | 0.842 | (-0.868, 0.709) |  | -0.006 | 0.20 | (-0.016, 0.003) |
| CCS | 132 | -0.002 | 0.29 | (-0.007, 0.002) |  | -0.070 | 0.175 | (-0.171, 0.031) |  | -0.0003 | 0.68 | (-0.001, 0.001) |
|  |  |  |  |  |  |  |  |  |  |  |  |  |
| NC group |  |  |  |  |  |  |  |  |  |  |  |  |
| MMSE | 66 | -0.023 | 0.002 | (-0.038, -0.008) |  | -0.510 | 0.001 | (-0.811, -0.210) |  | 0.007 | 0.001 | (0.003, 0.010) |
| MMSE orientation | 66 | -0.004 | 0.12 | (-0.009, 0.001) |  | -0.015 | 0.79 | (-0.130, 0.099) |  | 0.001 | 0.300 | (-0.001, 0.002) |
| FCSRT | 66 | 0.004 | 0.93 | (-0.084, 0.091) |  | -0.968 | 0.32 | (-2.904, 0.968) |  | 0.003 | 0.79 | (-0.021, 0.027) |
| DSST-WAISR | 66 | -0.011 | 0.82 | (-0.103, 0.081) |  | -0.993 | 0.16 | (-2.398, 0.412) |  | 0.006 | 0.50 | (-0.011, 0.023) |
| Category naming | 66 | -0.004 | 0.91 | (-0.066, 0.059) |  | -0.572 | 0.34 | (-1.773, 0.629) |  | 0.005 | 0.54 | (-0.010, 0.019) |
| CCS | 66 | -0.002 | 0.53 | (-0.008, 0.004) |  | -0.065 | 0.31 | (-0.192, 0.062) |  | 0.0004 | 0.56 | (-0.001, 0.002) |
|  |  |  |  |  |  |  |  |  |  |  |  |  |
| MCI group |  |  |  |  |  |  |  |  |  |  |  |  |
| MMSE | 66 | 0.007 | 0.30 | (-0.006, 0.019) |  | -0.025 | 0.89 | (-0.389, 0.339) |  | -0.002 | 0.29 | (-0.007, 0.002) |
| MMSE orientation | 66 | 0.001 | 0.66 | (-0.004, 0.007) |  | -0.108 | 0.50 | (-0.004, 0.007) |  | -0.001 | 0.61 | (-0.005, 0.003) |
| FCSRT | 66 | -0.025 | 0.53 | (-0.105, 0.054) |  | -2.995 | 0.001 | (-4.768, -1.222) |  | 0.017 | 0.10 | (-0.003, 0.038) |
| DSST-WAISR | 66 | -0.076 | 0.01 | (-0.135, -0.017) |  | -1.636 | 0.01 | (-2.824, -0.447) |  | 0.011 | 0.13 | (-0.003, 0.025) |
| Category naming | 66 | 0.027 | 0.28 | (-0.022, 0.076) |  | 0.217 | 0.67 | (-0.788, 1.223) |  | -0.015 | 0.01 | (-0.027, -0.003) |
| CCS | 66 | -0.001 | 0.55 | (-0.006, 0.003) |  | -0.122 | 0.11 | (-0.273, 0.029) |  | -0.0004 | 0.66 | (-0.002, 0.001) |

FCSRT: Free and cued selective reminding test; DSST-WAISR: Digit Symbol Substitution Test score from the Wechsler Adult Intelligence Scale—Revised

Table 6 Mixed-effects linear analysis of plasma NfL with cognitive functions in stratified NfL quartile subgroups (analysis on MAPT control group only)

|  | Sample size | NfL+ group^#^ | | |  | Time | | |  | NfL+ group^#^ × Time | | |
| --- | --- | --- | --- | --- | --- | --- | --- | --- | --- | --- | --- | --- |
|  |  | Coefficient | p | 95% CI |  | Coefficient | p | 95% CI |  | Coefficient | p | 95% CI |
| NC group |  |  |  |  |  |  |  |  |  |  |  |  |
| MMSE | 66 | -1.51 | 0.01 | (-2.60, -0.43) |  | 0.22 | 0.11 | (-0.05, 0.49) |  | 0.28 | 0.06 | (-0.01, 0.58) |
| MMSE orientation | 66 | -0.09 | 0.64 | (-0.45, 0.28) |  | 0.05 | 0.29 | (-0.05, 0.15) |  | 0.02 | 0.75 | (-0.09, 0.12) |
| FCSRT | 66 | -0.12 | 0.97 | (-6.36, 6.12) |  | 0.09 | 0.91 | (-1.49, 1.67) |  | 0.97 | 0.27 | (-0.75, 2.69) |
| DSST-WAISR | 66 | -5.42 | 0.10 | (-11.86, 1.02) |  | 0.14 | 0.81 | (-1.02, 1.29) |  | 0.83 | 0.20 | (-0.43, 2.08) |
| Category naming | 66 | -0.04 | 0.99 | (-4.53, 4.45) |  | 0.01 | 0.99 | (-1.00, 1.01) |  | 0.28 | 0.61 | (-0.81, 1.38) |
| CCS | 66 | -0.18 | 0.39 | (-0.59, 0.23) |  | 0.01 | 0.92 | (-0.10, 0.11) |  | 0.04 | 0.46 | (-0.07, 0.16) |
|  |  |  |  |  |  |  |  |  |  |  |  |  |
| MCI group |  |  |  |  |  |  |  |  |  |  |  |  |
| MMSE | 66 | -0.29 | 0.58 | (-1.32, 0.74) |  | -0.31 | 0.07 | (-0.64, 0.03) |  | -0.14 | 0.46 | (-0.50, 0.23) |
| MMSE orientation | 66 | 0.12 | 0.60 | (-0.33, 0.57) |  | -0.30 | 0.03 | (-0.57, -0.03) |  | -0.15 | 0.32 | (-0.46, 0.15) |
| FCSRT | 66 | -4.12 | 0.21 | (-10.58, 2.34) |  | -1.27 | 0.12 | (-2.89, 0.35) |  | 0.50 | 0.58 | (-1.30, 2.31) |
| DSST-WAISR | 66 | -6.79 | 0.01 | (-11.63, -1.95) |  | -0.34 | 0.53 | (-1.40, 0.73) |  | 0.60 | 0.32 | (-0.58, 1.79) |
| Category naming | 66 | 1.25 | 0.54 | (-2.79, 5.30) |  | -1.79 | <0.001 | (-2.71, -0.87) |  | -1.07 | 0.04 | (-2.08, -0.05) |
| CCS | 66 | -0.19 | 0.31 | (-0.55, 0.17) |  | -0.24 | <0.001 | (-0.37, -0.10) |  | -0.11 | 0.16 | (-0.25, 0.04) |

^#^ Participants in the non-upper quartile (NfL-) group as the reference

FCSRT: Free and cued selective reminding test; DSST-WAISR: Digit Symbol Substitution Test score from the Wechsler Adult Intelligence Scale—Revised

**Description of exploratory analysis using a structural equation modeling (SEM)**

Our study has identified two cognitive scores (i.e., DSST-WAISR and CCS) that were significantly associated with plasma NfL in both the whole population and the MCI group. We hypothesized that plasma NfL might have both direct and indirect correlations with cognitive function while the indirect correlation is mediated by cognition-related brain structures. Therefore, confirmatory factor analysis (CFA) was performed using SEM to explore these hypotheses.

**Model construction**

The model was based on 176 participants (85 MCI participants) with available data. A latent variable of brain degeneration was created by baseline MRI data (i.e., white matter volume, white matter hyperintensities, hippocampal volume and amygdala volume). To create a consistent direction of associations between these component variables with NfL, we converted the data of white matter, hippocampal and amygdala volumes to negative values based on the assumptions that the decreases of these volumes were associated with cognitive decline and increased plasma NfL value.

Direct effects of brain degeneration, plasma NfL, MAPT group, age and BMI were included in the SEM model together with an indirect effect of plasma NfL mediated by brain degeneration (Figure 1).

Figure 1. Path diagrams of structural equation modeling


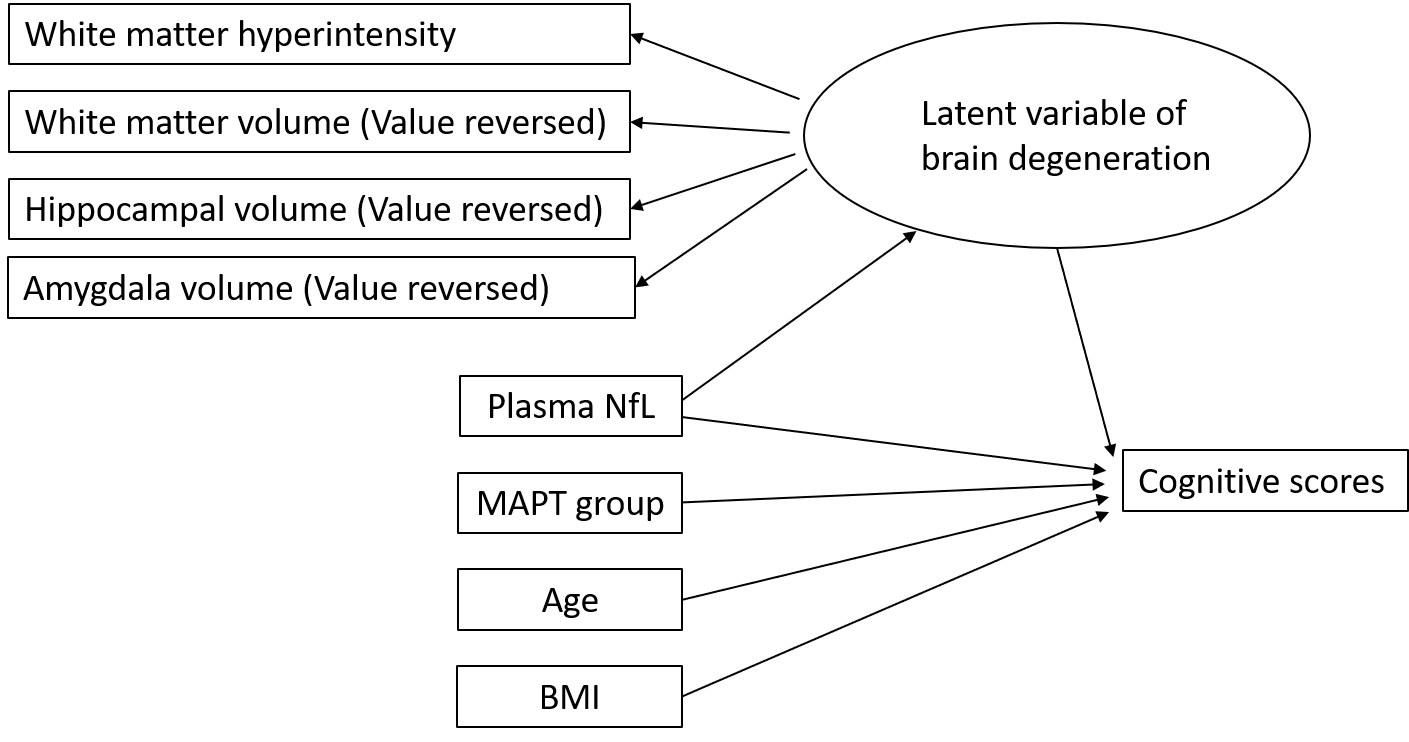


**Results**

The results of models based on the whole population showed significant direct effects of plasma NfL on cognitive scores while the indirect effects mediated by cognition-related brain structures were not significant (Table 1). Similar results were found regarding the WAISR score based on the MCI population while no direct or indirect effects were found in the CCS score (Table 2).

Table 7 Results of models based on the whole population (n = 176)

| Cognitive score | CCS | | |  | DSST-WAISR | | |
| --- | --- | --- | --- | --- | --- | --- | --- |
|  | Estimate | Std. Err | p |  | Estimate | Std. Err | p |
| Latent variable model |  |  |  |  |  |  |  |
| Brain degeneration ~ |  |  |  |  |  |  |  |
| White matter hyperintensity | 1 |  |  |  | 1 |  |  |
| Reversed hippocampal volume | 0.056* | 0.024 | 0.021 |  | 0.057* | 0.024 | 0.019 |
| Reversed amygdala volume | 0.045* | 0.02 | 0.021 |  | 0.043* | 0.018 | 0.019 |
| Reversed white matter volume | -0.112 | 0.098 | 0.255 |  | -0.102 | 0.095 | 0.282 |
| Regression model |  |  |  |  |  |  |  |
| Brain degeneration ~ |  |  |  |  |  |  |  |
| NfL | 0.108* | 0.051 | 0.034 |  | 0.11* | 0.051 | 0.031 |
| Cognitive score ~ |  |  |  |  |  |  |  |
| Brain degeneration | 0.009 | 0.006 | 0.168 |  | 0.138 | 0.103 | 0.179 |
| NfL | -0.003* | 0.001 | 0.014 |  | -0.052* | 0.023 | 0.025 |
| Age | -0.055** | 0.011 | <0.001 |  | -0.635** | 0.181 | <0.001 |
| BMI | -0.004 | 0.012 | 0.7 |  | -0.308 | 0.19 | 0.106 |
| MAPT group: Multidomain plus omega-3 | 0.058 | 0.12 | 0.63 |  | -0.261 | 1.975 | 0.895 |
| MAPT group: Omega-3 | 0.296* | 0.133 | 0.026 |  | 0.853 | 2.191 | 0.697 |
| MAPT group: Multidomain | 0.098 | 0.13 | 0.451 |  | 1.558 | 2.149 | 0.469 |
| SRMR | 0.086 | | |  | 0.08 | | |
| χ2 | 103.717 | | |  | 97.456 | | |
| CFI | 0.656 | | |  | 0.65 | | |
| GFI | 0.822 | | |  | 0.83 | | |
| AGFI | 0.581 | | |  | 0.599 | | |

*: p < 0.05, **: p < 0.01

SRMR: standardized root mean square residual; CFI: comparative fix index; GFI: goodness-of-fit index; AGFI: adjusted goodness-of-fit index

Table 8 Results of models based on the MCI population (n = 85)

| Cognitive score | CCS | | |  | DSST-WAISR | | |
| --- | --- | --- | --- | --- | --- | --- | --- |
|  | Estimate | Std. Err | p |  | Estimate | Std. Err | p |
| Latent variable model |  |  |  |  |  |  |  |
| Brain degeneration ~ |  |  |  |  |  |  |  |
| White matter hyperintensity | 1 |  |  |  | 1 |  |  |
| Reversed hippocampal volume | 0.046* | 0.02 | 0.018 |  | 0.048* | 0.022 | 0.029 |
| Reversed amygdala volume | 0.037* | 0.016 | 0.019 |  | 0.045* | 0.021 | 0.032 |
| Reversed white matter volume | -0.007 | 0.105 | 0.945 |  | -0.028 | 0.115 | 0.805 |
| Regression model |  |  |  |  |  |  |  |
| Brain degeneration ~ |  |  |  |  |  |  |  |
| NfL | 0.16* | 0.075 | 0.032 |  | 0.14* | 0.071 | 0.05 |
| Cognitive score ~ |  |  |  |  |  |  |  |
| Brain degeneration | 0.006 | 0.006 | 0.378 |  | 0.204 | 0.133 | 0.123 |
| NfL | -0.002 | 0.002 | 0.212 |  | -0.06* | 0.03 | 0.044 |
| Age | -0.058** | 0.016 | <0.001 |  | -0.825** | 0.239 | 0.001 |
| BMI | 0.005 | 0.017 | 0.764 |  | -0.292 | 0.257 | 0.257 |
| MAPT group: Multidomain plus omega-3 | 0.105 | 0.161 | 0.514 |  | 1.621 | 2.463 | 0.51 |
| MAPT group: Omega-3 | 0.13 | 0.199 | 0.514 |  | -4.779 | 3.029 | 0.115 |
| MAPT group: Multidomain | -0.217 | 0.184 | 0.238 |  | -2.581 | 2.811 | 0.359 |
| SRMR | 0.108 | | |  | 0.109 | | |
| χ2 | 84.627 | | |  | 84.407 | | |
| CFI | 0.584 | | |  | 0.578 | | |
| GFI | 0.744 | | |  | 0.746 | | |
| AGFI | 0.397 | | |  | 0.401 | | |

*: p < 0.05, **: p < 0.01

SRMR: standardized root mean square residual; CFI: comparative fix index; GFI: goodness-of-fit index; AGFI: adjusted goodness-of-fit index
